# Prognostic evaluation of system immune-inflammatory index and prognostic nutritional index in double expressor diffuse large B-cell lymphoma

**DOI:** 10.1515/med-2023-0819

**Published:** 2023-10-17

**Authors:** Fang Su, Ke Lian

**Affiliations:** Department of Epidemic, Shanxi Province Cancer Hospital/Shanxi Hospital Affiliated to Cancer Hospital, Chinese Academy of Medical Sciences/Cancer Hospital Affiliated to Shanxi Medical University, Taiyuan, 030013, China; Department of Oncology, Shanxi Bethune Hospital, Shanxi Academy of Medical Sciences Tongji Shanxi Hospital, Third Hospital of Shanxi Medical University, Taiyuan, 030032, China

**Keywords:** diffuse large B-cell lymphoma, double expressor lymphoma, SII, prognostic nutritional index

## Abstract

Predicting MYC and BCL2 double-expressor lymphoma prognosis using the system immune-inflammatory index (SII) and prognostic nutritional index (PNI) (DEL). From January 2015 to December 2021, 281 diffuse large B-cell lymphoma (DLBCL) wax blocks were used to make tissue chips. Screening double expressor lymphoma (DEL) instances involved immunocytochemistry and fluorescence in situ hybridization. Academic analysis used clinicopathological characteristics and follow-up data. SII, PNI, and DEL prognosis were correlated using univariate and multivariate cox regression analysis. The median age of 78 DEL patients is 60 (range: 43–74). SII and PNI cut-off values of 603.5, 3.07, and 144 predict PFS and OS well. Lower SII is associated with longer PFS (HR for SII = 0.34, 95% CI 0.15–0.76, *P* = 0.006; HR for NLR = 0.46, 95% CI 0.22–0.99, *P* = 0.048; HR for PLR = 0.39, 95% CI 0.17–0.94, *P* = 0.025; LMR = 0.39, 95%, CI 0.17–0.94, *P* = 0.025) and OS (HR for SII = 0.16, 95% CI 0.05–0.51, *P* = 0.005; HR for PNI = 0.20, 95% CI 0.06–0.62, *P* = 0.002). SII and PNI are promising predictors for twofold expressor DLBCL. Combining these increase prediction accuracy.

## 1 Introduction

Diffuse large B-cell lymphoma (DLBCL) is highly aggressive and heterogeneous [1,2], and there are still many deficiencies in accurate diagnosis and treatment. Double expressor lymphoma (DEL) accounts for 19–34% of DLBCL [3–5], and Myc expression ≥40% and Bcl-2 expression ≥50% in immunohistochemistry were used as criteria, but there are differences in the cut-off values defined by different studies [6–8]. Since the diagnostic criteria (MYC and BCL-2 positive thresholds) and the prognostic relevance of DEL remain controversial and there is a lack of uniform and effective treatment options, DEL is currently not classified as a separate diagnostic type [9].

However, DEL is characterized by aggressiveness and a low response rate to the interventions used in the first treatment; it still has a poor overall prognosis and is prone to develop relapsed or refractory diffuse large B-cell lymphoma (R/R DLBCL) [10]. According to one study [11], DEL is also heterogeneous; some patients with DEL can achieve remission after treatment, and their prognosis treatment result is not different from non-DEL patients (DEL patients are still needed to screen out the truly high-risk patients while also reducing overtreatment of low-risk patients).

At present, there is no comprehensive understanding of the prognostic factors affecting DEL patients, and the correlation between the systemic immune-inflammation index (SII) and the prognostic nutritional index (PNI) and the prognosis of DEL has not been reported yet. In this study, we collected a group of newly treated DEL cases and analyzed the influence of SII and PNI on the prognosis of DEL through immunohistochemistry and chemical staining, clinicopathological data collection, and follow-up, in order to provide the basis for treatment and further stratify the diagnosis and treatment of DEL cases.

## 2 Materials and methods

This retrospective study was conducted at the Department of Oncology performed from January 2015 to December 2021. The Institutional Review Board and the local ethical committee have approved the study after registration with the Research Centre of Shanxi Medical University (protocol SMU # RC/IRB/2015/1067). This study followed the criteria as according to declaration. All participants signed and provided written informed consent after a full explanation of the process’s procedure and safety. According to the set criteria, those who were not ready or failed to report were excluded from the study.

### 2.1 Patient characteristics

#### 2.1.1 Retrospective analysis

**Figure 1:**
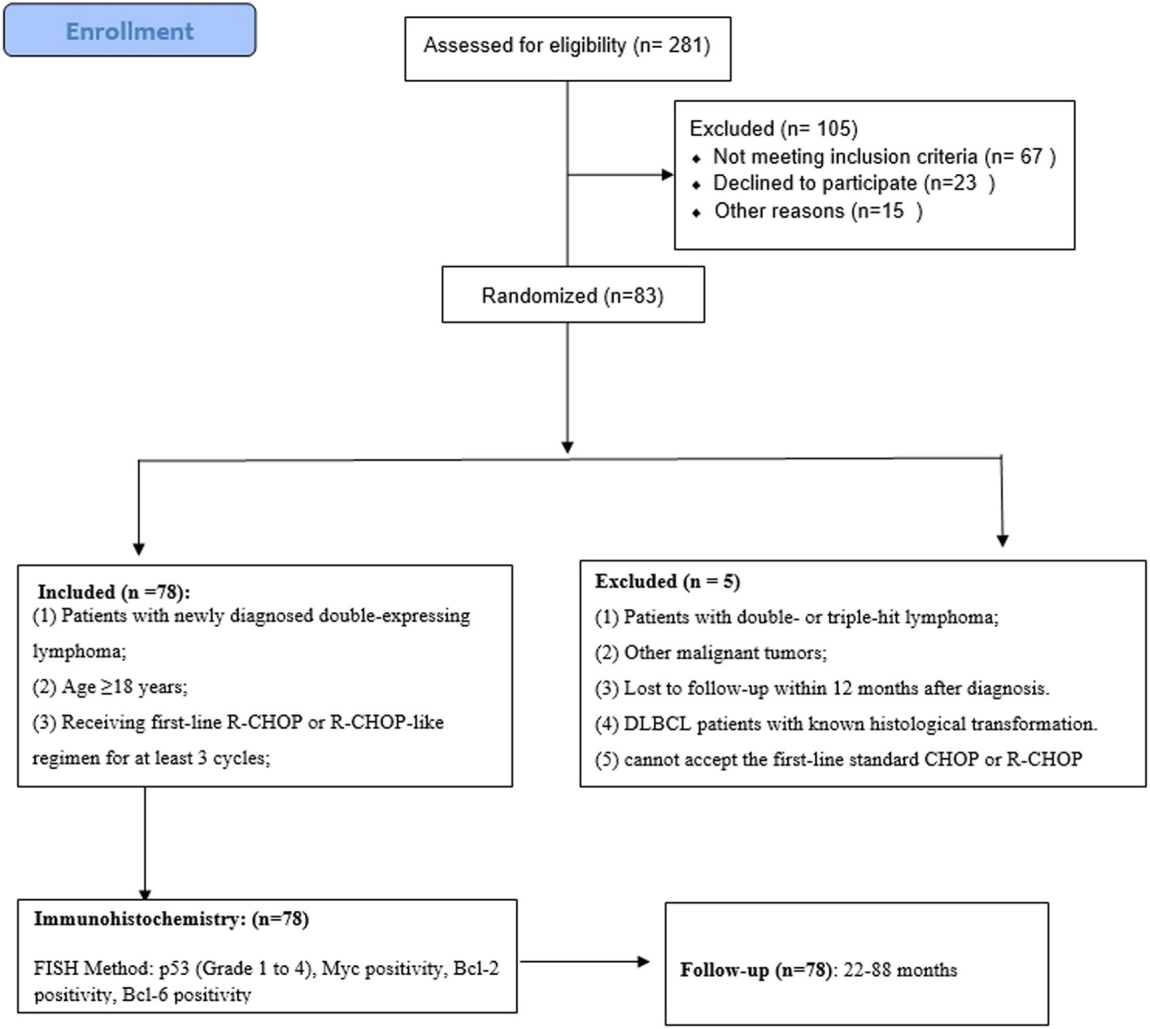
Study flow diagram: immunohistochemistry.

Between January 2015 and December 2021, 281 patients with histopathology-diagnosed DLBCL underwent surgical removal in our hospital, underwent a second examination by two expert pathologists, and selected the appropriate wax blocks to create tissue chips. The corresponding markers were detected by immunohistochemistry and fluorescence in situ hybridization (FISH), and a total of 83 DEL cases were screened, of which 5 were lost to follow-up and 78 patients had complete data. Inclusion criteria are: (1) patients with newly diagnosed double-expressing lymphoma, (2) age 18 years or older, (3) receiving a first-line R-CHOP or R-CHOP-like regimen for at least three cycles, and (4) complete medical records and follow-up data. Exclusion criteria: (1) patients with double- or triple-hit lymphoma confirmed by FISH in pathological tissue, (2) combining other malignant tumors, (3) lost to follow-up within 12 months after diagnosis, (4) DLBCL patients with known histological transformations, and (5) those who cannot accept the first-line standard CHOP or R-CHOP regimen due to infection, poor physical fitness, etc. (Figure 1).

The complete DLBCL paraffin tissue preserved in the pathology department was collected, and serial sections with a thickness of 4 μm were prepared for immunohistochemical detection. The clinical data of patients was collected, including gender, age, clinical stage, ECOG score, and absolute value of neutrophils, absolute value of lymphocytes, platelets, and hemoglobin levels.

#### 2.1.2 Calculations

The optimal cut-off values for neutrophil-to-lymphocyte ratio (NLR), platelet-to-lymphocyte ratio (PLR), lymphocyte count/monocyte count (LMR), and SII were calculated using the receiver operating characteristic (ROC) curve as follows: NLR = absolute value of neutrophils/absolute value of lymphocytes; PLR = absolute value of platelets/absolute value of lymphocytes, LMR = absolute value of lymphocytes/absolute value of monocytes, SII = absolute number of platelet × absolute number of neutrophil/absolute value of lymphocytes, PNI = 10 × serum albumin (g/dL) + 0.005 × lymphocyte count/µL. In order to enhance the precision and consistency of DEL prognostic prediction, two additional factors, namely COSII and PNI, were included.

### 2.2 Immunohistochemical staining and interpretation of results

Antibodies such as Myc, Bcl2, Bcl-6, CD10, MUM-1, Ki-67, and P53 were purchased from Beijing Zhongshan Golden Bridge Biotechnology Co., Ltd, and related reagents such as secondary antibodies were purchased from Ventana Medical Systems, United States. A Roche Bench Mark XT automated immunohistochemistry instrument was applied, using the EnVision two-step method. Myc proteins are localized in the nucleus and are classified according to the percentage of positive cells as grade 0 (0), grade 1 (1–25%), grade 2 (26–50%), grade 3 (51–75%), and grade 4 (76–100%) [12]. Bcl-2 positivity was defined as the presence of brownish-yellow granules in the cytoplasm/membrane of ≥50% of tumor cells. Bcl-6 positivity was defined as the presence of brownish-yellow granules in the nucleus of ≥50% of tumor cells [13,14]. CD10 positivity is defined as the presence of brownish-yellow granules in the cytoplasm of ≥30% of tumor cells and MUM-1 positivity is defined as the presence of brownish-yellow granules in the nucleus of ≥30% of tumor cells. According to the number of positive cells, P53 is graded as grade 0 (no positive cells), grade 1 (5%), grade 2 (6–10%), grade 3 (11–40%), and grade 4 (41%). Grades 2–4 are considered overexpression [15]. The above test results were re-interpreted by two experienced lymphopoietic pathologists for all sections to obtain accurate immunohistochemical results. In addition, immunohistochemical results of MYC, BCL-2, CD10, BCL-6, and MUM-1 proteins were analyzed by using the Hans method [16], and COO typing was performed to determine the germinal center B-cell-like (GCB) and non-GCB subtypes.

#### 2.2.1 The FISH method

The tumor areas were selected according to HE staining, and C-MYC, BCL-2, and BCL-6 gene disruption probes (all purchased from Guangzhou LBP Medicine Science & Technology Co., Ltd) were used for the experiments. The experimental procedures were carried out strictly according to the kit instructions. Signals from at least 100 tumor nuclei were recorded by using the Zeiss Axioimager fluorescence microscope at high power (100×). The probes are composed of two parts: a red fluorescent probe and a green fluorescent probe. Normal cells exhibit two yellow fusion signals or two adjacent red and green signals, whereas gene translocation exhibits one yellow signal and two adjacent red and green separation signals. When the proportion of isolated signals is greater than 10%, it is considered positive, and three yellow fusion signals in the same nucleus are considered multiple gene copies.

#### 2.2.2 Follow-up

The follow-up methods were telephone and outpatient follow-up methods until December 31, 2021. The follow-up time was from the date of diagnosis to the end of follow-up or the date of death. The median follow-up was 52 (22–88) months. Five of the 83 patients (6%) were lost to follow-up. Patients lost to follow-up were included in the survival analysis as censored data. Progression-free survival (PFS) time was defined as the time from diagnosis to relapse, patient death, or the end of follow-up; overall survival (OS) time was from diagnosis to the end of follow-up, and death was calculated from diagnosis to death.

### 2.3 Statistical analysis

Statistical analysis was performed using SPSS 24.0 software. Univariate analysis of variance and multivariate cox regression analysis were used to explore the correlation between SII, PNI, and DEL prognosis. The risk factors for DEL were investigated using univariate and multivariate logistic regression analysis. The model prediction accuracy is calculated as (true negative + true positive)/total number of subjects.


**Ethical approval:** All procedures performed in studies involving human participants were in accordance with the ethical standards of the institutional and/or national research committee and with the 1964 Helsinki declaration and its later amendments or comparable ethical standards.
**Informed consent:** Informed consent was obtained from all individual participants included in the study.

## 3 Results

### 3.1 Clinical and pathological features of patients

There were a total of 78 patients, and the median age was about 67 (21–89) years, of which 44 (69.8%) were ≥60 years; there were 40 males (51.2%); 33 (42.3%) had intranodal lymphoma, 18 (28.6%) had Ann Arbor stage I–II, and 45 (71.4%) had Ann Arbor stage III–IV; 21 (33.3%) had pathological GCB and 42 (66.7%) had non-GCB; 16 (25.4%) had B symptoms; 29 (46%) had an ECOG score of 2; and 24 (38%) had two extra-nodal involvements. Fifty-eight had more than three NCCN-IPI, 12 (19%) had large masses (>7.5 cm), 30 cases (47.6%) had elevated lactate dehydrogenase (LDH) levels, 31 (49.2%) had β2-MG exceeding the upper limit of normal, and 31 (49.2%) had β2-MG exceeding the upper limit of normal. Fifty cases (79.4%) had serum albumin <40 g/L), 12 (19%) had P53 grades 1–2 and 51 (81%) had grades 3–4, 3 (4.8%) had Myc positivity, 0 (0%) had Bcl-2 positivity), and 6 (9.5%) had Bcl-6 positivity in the FISH test (Table 1).

**Table 1:** Patient demographics and baseline evaluation

Clinical feature		Numbers	*n* (%)
Age	≥60	44	69.8
	<60	34	30.2
Gender	M	40	51.2
	F	38	26.8
Part	Intranodal lymphoma	33	42.3
	Extranodal lymphoma	45	71.4
Ann Arbor stage	I–II	18	28.6
	III–IV	45	71.4
Cellular origin	GCB	21	33.3
	Non-GCB	57	66.7
B symptoms	Yes	16	25.4
	No	62	74.6
ECOG	≥3	29	46
	<2	71	54
NCCN-IPI	≤3	20	25.7
	>3	58	74.3
Large lumps	Yes	12	19
	No	54	59
LDH	≤250	30	47.6
	>250	48	52.4
B2-MG	≤1.8	31	49.2
	>1.8	31	49.2
Serum albumin	<40 g/L	50	79.4
	>40 g/L	28	20.6
**FISH test**			
P53	Grade 1–2	12	19
	Grade 3–4	51	81
Myc positivity	Positive	3	4.8
Bcl-2 positivity	Positive	0	0
Bcl-6 positivity	Positive	6	9.5

Note: NCCN-IPI: improved International Prognostic Index; LDH: lactate dehydrogenase; GCB: germinal center; non-GCB: non-germinal center.

### 3.2 Determination and grouping of boundary values of NLR, PLR, LMR, SII, and PNI

**Figure 2:**
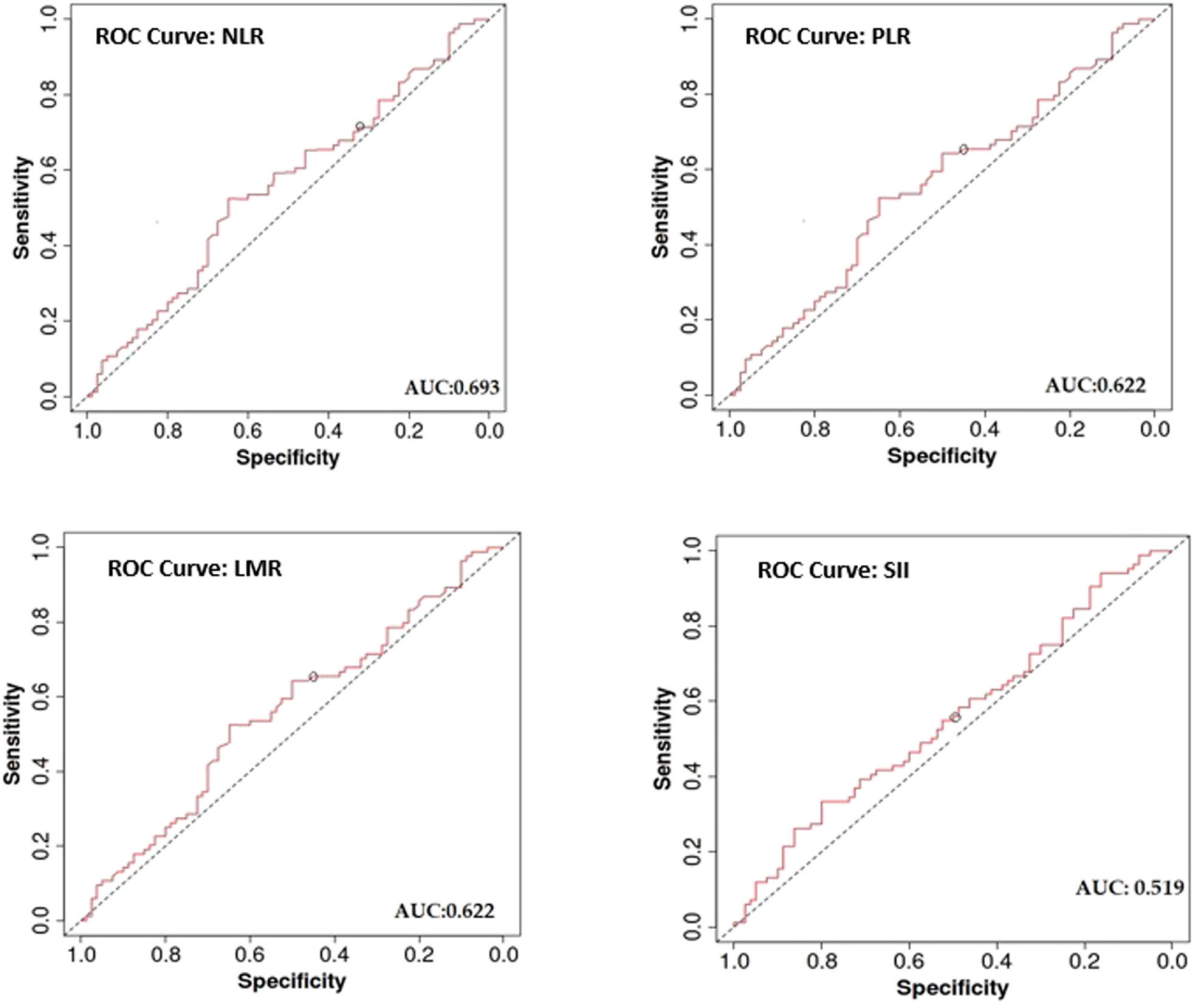
ROC curves: optimal NLR, PLR, LMR, and SII cut-off values.

Based on the ROC curve (Figure 2), with PFS and OS as study endpoints, the optimal NLR, PLR, LMR, and SII cut-off values were computed as 1.73 (AUC: 0.693, sensitivity: 0.483, specificity: 0.853), 306.494 (AUC: 0.622, sensitivity: 0.897, specificity: 0.382), 4.867 (AUC: 0.622, sensitivity: 0.862, specificity: 0.412), and 304.2 (AUC: 0.519, sensitivity: 0.345, specificity: 0.794).

### 3.3 Relationship between SII, PNI, and PFS in DEL patients

The relationship between SII, PNI, and PFS in DEL patients was analyzed via Cox univariate and multivariate analysis (Table 2). The grouping was low SII–high PNI, high SII–low PNI, low SII–low PNI, and high SII–high PNI. Considering that SII and PNI are two-dimensional composite parameters, they have their own predictive value. To improve the accuracy and stability of DEL prognosis prediction, we further combined these two parameters, namely COSII and PNI. COSII indicators are PCR-based markers derived from single-copy conserved orthologous genes, and PNI is prognostic nutritional index. Elevated SII and decreased PNI were recorded as 0, simultaneous increases or decreases in SII and PNI were recorded as 1, and decreased SII and elevated PNI were recorded as 2. By Kaplan-Meier curve calculation and the log-rank test, DELT patients were divided into three groups according to their COSII–PNI value (*P* < 0.001). We found that the prognosis of patients in the COSII–PNI = 1 and COSII–PNI = 2 subgroups was better than that in the COSII–PNI = 0 subgroup (*P* < 0.001).

**Table 2:** One-way analysis of variance and chi-square test of influencing factors of DEL prognosis (PFS)

Variable	Adverse factors	One-way ANOVA		Chi-square test	
		Eta	*p*	*x* ^2^	*p*
Age	≥60	0.003	0.001	21.14	0.024
Gender	M	0.003	0.015	8.95	0.001
Part	Extranodal lymphoma	0.024	0.002	17.01	0.016
Origin	non-GCB	0.006	0.034	10.23	0.003
P53	≥50%	0.042	0.002	21.13	0.022
Ki67	≥80%	0.005	0.004	16.23	0.015
LDH (IU/L)	≥250	0.047	0.035	34.12	0.037
Serum albumin (g/L)	≥40	0.035	0.004	26.54	0.025
Stage	III, IV	0.025	0.003	32.33	0.033
β2-MG	≥3	0.035	0.018	45.12	0.045
Extranodal amount	≥2	0.059	0.026	22.53	0.019
ECOG	≥2	0.002	0.037	19.15	0.012
NLR	≥1.73	0.001	0.001	11.54	0.011
PLR	≥306.494	0.015	0.039	10.68	0.018
SII	≥304.2	0.021	0.022	9.56	0.012
LMR	≥4.867	0.018	0.014	31.98	0.031
Origin	Non-GCB	0.034	0.031	44.21	0.044
B symptoms	Yes	0.027	0.005	21.59	0.021
Large lumps	Yes	0.004	0.021	8.67	0.001

## 4 Discussion

With the promotion and popularity of R-CHOP regimens, the cure rate of DLBCL has reached 60%, but there are still 30–40% of patients who present with initial refractory or relapse resistance [4,6], and it is a current research hotspot on how to screen this group of patients more effectively. Studies found that the double expression status was a poor prognosis factor that was independent of the IPI index in DLBCL [5,7,16]. However, some DEL patients can be relieved after treatment, and the prognosis is not different from that of non-DEL patients. As a result of DEL’s heterogeneity, it is necessary to screen out truly high-risk patients in newly diagnosed DEL patients in order to provide a theoretical basis for precise treatment.

The previous studies have reported that there are several poor prognostic factors in patients with DEL, including elderly age, late disease stage, poor functional status, multiple extra-nodal sites, a high proliferation index [17], and elevated serum LDH [18]. With the increasing understanding of the disease, new prognostic factors have been reported, such as P53 protein overexpression [8,19–21] and CD5 protein overexpression [22,23]. The indicators reflecting systemic inflammatory responses are NLR, PLR, LMR, SII, etc. Clinical prognostic significance is rarely reported.

In an inflammatory response, especially in the process of chronic inflammation, the tumor microenvironment composed of inflammatory cells, inflammatory cytokines, and inflammatory signaling molecules participates in tumor growth, infiltration, and metastasis through complex regulatory mechanisms [24,25]. Based on these studies, some scholars propose to use inflammation-related markers as evaluation indicators for tumor disease assessment and prognosis prediction [24]. In 2014, Hu et al. [24] first defined SII as a new immune evaluation index based on a study on the prognosis of patients with hepatocellular carcinoma, and its formula is SII = *P* × *N*/*L* (*P*, *N*, *L* platelet count, neutrophil count, and lymphocyte count in routine blood tests). Subsequent studies have also confirmed that SII is expected to become an independent prognostic factor for tumor patients after surgery, which can help with the stratified management of risk groups. Ren et al. [26] examined the relationship between the preoperative SII and the PNI and the prognosis of patients with alveolar hydatid disease in 2021. SII and PNI can be regarded as independent risk factors that indicate the prognosis of patients with hepatic alveolar echinococcosis. Patients have a more favorable prognosis when their preoperative PNI and SII are greater and lesser, respectively. In addition, the concurrent use of SII and PNI prior to surgery has the potential to improve the accuracy of prognostication. The prognostic value of the SII and the PNI in medulloblastoma patients undergoing surgical resection was reported by Zhu et al. [27] in 2021. They emphasized the relationship between progression of cancer, metastasis, systemic immune inflammation, and nutritional dysfunction. They identified preoperative SII, albumin-bilirubin grade, and combined SII and PNI (COSII-PNI) as reliable prognostic indicators for patients undergoing surgical resection for malignant brain tumors.

## 5 Conclusion

Both the SII and the PNI are promising indicators for patients with double expressor DLBCL, and the combination of these two increases the accuracy of prediction.
